# Effect of macronutrient fertilization on olive oil composition and quality under irrigated, intensive cultivation management

**DOI:** 10.1002/jsfa.12110

**Published:** 2022-07-16

**Authors:** Isaac Zipori, Uri Yermiyahu, Arnon Dag, Ran Erel, Alon Ben‐Gal, Liu Quan, Zohar Kerem

**Affiliations:** ^1^ Agricultural Research Organization – Volcani Institute, Gilat Research Center Israel; ^2^ College of Agronomy, Sichuan Agricultural University Chengdu China; ^3^ Institute of Biochemistry, Food Science and Nutrition, The Robert H. Smith Faculty of Agriculture, Food and Environment The Hebrew University of Jerusalem Rehovot Israel

**Keywords:** olive, fertilization, oil quality, nitrogen, potassium, phosphorus

## Abstract

**BACKGROUND:**

Intensive olive (*Olea europaea* L.) orchards are fertilized, mostly with the macronutrients nitrogen (N), phosphorus (P) and potassium (K). The effects of different application levels of these nutrients on olive oil composition and quality were studied over 6 years in a commercial intensively cultivated ‘Barnea’ olive orchard in Israel.

**RESULTS:**

Oil quality and composition were affected by N, but not P or K availability. Elevated N levels increased free fatty acid content and reduced polyphenol level in the oil. Peroxide value was not affected by N, P or K levels. The relative concentrations of palmitoleic, linoleic and linolenic fatty acids increased with increasing levels of N application, whereas that of oleic acid, monounsaturated‐to‐polyunsaturated fatty acid ratio and oleic‐to‐linoleic ratio decreased.

**CONCLUSION:**

These results indicate that intensive olive orchard fertilization should be carried out carefully, especially where N application is concerned, to avoid a decrease in oil quality due to over‐fertilization. Informed application of macronutrients requires leaf and fruit analyses to establish good agricultural practices, especially in view of the expansion of olive cultivation to new agricultural regions and soils. © 2022 The Authors. *Journal of The Science of Food and Agriculture* published by John Wiley & Sons Ltd on behalf of Society of Chemical Industry.

## INTRODUCTION

Over the last few decades, the oil olive industry has been shifting from non‐irrigated, extensive olive orchard management to irrigated, intensive practices.^1^ Introduction of the latter includes the installation of irrigation and fertilization systems^2^, ^3^ as well as the adaptation of new irrigation and fertilization practices that affect both yield and oil quality.^1^, ^4^, ^5^ Olive (*Olea europaea* L.) oil is a major component of the Mediterranean diet^6^ and is highly appreciated for its protective effects against illnesses, such as coronary heart disease, some types of cancer and age‐related cognitive decline.^7^, ^8^

One of the main health‐related attributes of olive oil is its high levels of monounsaturated fatty acids (MUFA) – especially oleic acid – which comprises 55–85% of its fatty acid content. The health‐promoting properties of oleic acid have been widely studied, and its benefits have been established. For instance, a recent systematic review and meta‐analysis suggested that oleic acid supplementation exerted a significant reduction in the concentration of C‐reactive proteins in the blood.^9^ Recently, it was suggested that olive oil or oleic acid consumption is as good as other strategies to manage the metabolic syndrome.^10^ Moreover, there is a fraction of microconstituents, such as the two essential polyunsaturated fatty acids (PUFA) linoleic (C18:2 omega‐6) and linolenic (C18:3 omega‐3), phytosterols, squalene, tocopherols and last, and certainly not the least, phenolic compounds (polyphenolics, PP), which are widely recognized for their contribution to the health of consumers.^11^, ^12^, ^13^, ^14^ Brouwer *et al*.^15^ found that high dietary amounts of α‐linolenic acid reduce the risk of coronary disease but increase that of prostate cancer. Cho *et al*.^16^ found that high dietary levels of linolenic acid can increase the risk of age‐related macular degeneration. However, a human dietary ratio of 10:1 for omega‐6 to omega‐3 fatty acids is recommended by the Merck Manual,^17^ which corresponds to the ratio of these fatty acids in olive oil. In addition to its possible nutritional importance, linolenic acid content is a parameter for determining the authenticity of olive oil in the Codex Alimentarius, with a maximum level set at 0.9%. The PP and tocopherols – natural bitter and pungent compounds that are often also considered as natural antioxidants (secoiridoid derivatives and vitamins E) – protect PUFA from oxidation^18^, ^19^ and positively contribute to consumer health.^9^, ^11^, ^20^, ^21^, ^22^, ^23^

Free fatty acid (FFA) content is a primary parameter for olive oil quality, determining its market value and its suitability for human consumption. Extra virgin olive oil (EVOO) is classified as such when its FFA content is less than 0.8%, whereas non‐edible olive oil contains above 3.3% FFA. The PP content of virgin olive oil (VOO) is another important quality parameter. These are strong antioxidants that have multiple roles in olive oil; in addition to protecting PUFA from oxidation, thereby extending the oil's shelf life, PP from the consumed oil serve as antioxidants in human tissues.^18^ The European Food Safety Authority (EFSA) regulation EU432/2012 states that ‘olive oil phenols contribute to the protection of blood lipids from oxidative stress,^24^, ^25^ when their level is above 250 mg kg^−1^. PP also affect the sensory properties of olive oil, determining its bitterness and pungency.

The increasing demand for high‐quality olive oil is an important factor in assessing the overall performance of olive orchards. Olive oil quality, especially FFA levels and PP content, but also fatty acid composition, is closely related to agricultural practices such as irrigation,^26^ timing of harvest^27^ and fertilization levels.^28^ Fernandez‐Escobar *et al*.^29^ found that over‐fertilization with N results in a decrease in PP content and the oil's oxidative stability. Morales‐Sillero *et al*.^30^ reported that elevated N–P–K levels lead to a similar response, but no distinction between the effects of specific nutrients was made in that study. In a controlled container experiment carried out at the Gilat Research Center in Israel, increasing N levels were negatively correlated with both FFA and PP contents, but had no effect on peroxide value (PV). Increasing P level was negatively correlated with PV and PP content, but had no effect on FFA, and increasing K levels had no effect on any of these parameters.^28^, ^31^, ^32^ Most of the available information regarding the effect of olive orchard fertilization on oil quality has been obtained from either extensive, non‐irrigated orchards^29^ or container experiments, where trees are often grown in substrate and not in soil.^31^ Little work on this subject has been published from experiments, reflecting conditions found in commercial‐scale, modern, intensive, irrigated orchards. In the current study, the effect on oil quality of different N, P and K fertilization levels, applied over 6 years to olive trees in a commercial olive orchard under Mediterranean conditions, is presented and discussed. Vegetative and reproductive performance of the trees from the same experiment is published in Haberman *et al*.^4^, ^5^

## MATERIALS AND METHODS

The experiment was carried out over 6 years (2011–2016) in a commercial intensively cultivated olive orchard located on the southern coastal plain of Israel at an elevation of 80 m above sea level, near Kibbutz Negba (31° 39′ 7.50″ N; 34° 40′ 54.00″ E). The average annual temperature and rainfall at the site were 20.1 °C and 490 mm, respectively. Orchard soil texture varied between loam and clay loam. The orchard was planted in 2007 with cv. Barnea trees,^33^ at 4 m × 7 m spacing, for a density of 360 trees ha^−1^. From 2007 until 2010, the orchard was alternately irrigated with brackish water and recycled wastewater according to water availability. From 2010, the orchard was irrigated with fresh water (electrical conductivity = 0.4–0.5 dS m^−1^). Trees were generally trained to form a cup shape suitable for mechanical trunk shaking, with a single trunk of about 70 cm in height and three to five leaders per tree. The orchard was monitored and treated regularly for pests, especially the olive fly (*Bactrocera oleae*). Experimental plots consisting of at least 12 trees (three rows, four trees per row) were allocated over 3 ha of the orchard. Two uniform trees in the center of each plot were selected for sampling and measurements. The experimental setup was randomized blocks with seven plots per treatment, giving 14 trees (replicates) per treatment. Trees were irrigated twice a week through a drip system (one drip line per row of trees with 1.3 L h^−1^ drippers spaced at 0.75 cm). Seasonal irrigation was initiated after three consecutive weeks without precipitation (usually during March–April) and stopped after the first significant rainfall (at least 20 mm, usually occurring in October–November). Irrigation quantity was according to local commercial practice, determined according to the return of potential evapotranspiration, calculated by a modification of the Penman–Monteith method,^34^ multiplied by a crop coefficient ranging within the season between 0.27 and 0.70. Meteorological data were obtained from a nearby meteorological station (Negba) of the Israeli Ministry of Agriculture and Rural Development. Annual irrigation amounts varied from 480 to 620 mm. Average winter precipitation was 487 mm, falling mainly from October to April. Fertilizers were applied continuously through the irrigation system (fertigation), starting at the beginning of the irrigation season and during each irrigation event until annual target application levels had been reached, at the end of August.

The experiment included four N, two P and two K fertilization levels. The amounts of each nutrient applied per treatment are specified in Table 1. The treatments were designed such that each tested the effect of only one variable; for example, the low N treatment (N_75_) received the commonly applied P and K levels (35 and 250 kg ha^−1^ y^−1^, respectively); the P_0_ treatment received no P, and 150 and 250 kg ha^−1^ y^−1^ of N and K, respectively, etc. The common commercial practice in the region is to apply 150, 35 and 250 kg ha^−1^ y^−1^ of N, P and K, respectively. In all treatments, the ratio of N forms was NH_4_^+^:NO_3_^−^ = 1:1.

**Table 1:** Fertilization treatments in the experiment. Tested variable: underlined and in bold letters

Treatment indication	Amount applied (kg ha^−1^ y^−1^)		
	N	P	K
N_0,40_	0 _(2011–2014) 40 (2015–2016)_	35	250
N_75_	75	35	250
N_150_	150	35	250
N_300_	300	35	250
	
	N	**P**	K
P_0_	150	0	250
P_35_	150	35	250
	
	N	P	**K**
K_0_	150	35	0
K_250_	150	35	250

Differential fertilization treatments were initiated in June 2011 and were implemented over a period of six consecutive years, up to and including the 2016 season. During the experiment, trees in the plots receiving the 0 kg N ha^−1^ y^−1^ (initially indicated as N_0_) treatment appeared to be in poor condition, exhibiting severe visual N‐deficiency symptoms. Therefore, in order not to lose the trees completely, in the 2015 and 2016 seasons, their N fertilization was adjusted to 40 kg ha^−1^ and the treatment was redesignated as N_0,40_. Each fertilization level (treatment) had a separate computer‐controlled and monitored irrigation and fertilization application system, including a tailored liquid fertilizer source container. The liquid fertilizers were prepared according to each treatment's specifications and supplied by Israel Chemicals Ltd (ICL, Tel Aviv, Israel). Each fertilizer was compiled such that annual application per hectare included, in addition to the specified N, P and K levels, 3.8 kg iron (Fe), 1.9 kg zinc (Zn), 940 g manganese (Mn), 140 g copper (Cu) and 100 g molybdenum (Mo). To ensure accuracy, irrigation solution was sampled from the drippers every 2 weeks, from two plots for each treatment, and nutrient concentrations were confirmed. In the season prior to the initiation of the experiment (2010), the orchard was fertigated constantly during the irrigation season with annual amounts of 250 kg N ha^−1^, 175 kg K ha^−1^ and no P.

### Fruit sampling

Fruit was harvested upon ripening (‘50% black’), between October and November, from individual trees by a mechanical trunk‐shaker with simultaneous rod beating. Fruit yield per tree was weighed and a 3 kg subsample was taken to the laboratory and kept in a cool environment, in aerated boxes. Oil was cold‐extracted from the fruit within 24 h of harvest using a laboratory mill (Abencor System mc2, Ingenieria y sistemas, Seville, Spain), according to the procedure described by Ben‐David *et al*.^35^ Oil and water contents of the olive paste were measured by a calibrated near‐infrared analysis system (OliveScan, Foss, Hilleroed, Denmark).^36^

### Olive oil analysis

FFA levels and PV were determined using International Organization for Standardization (ISO) analytical methods 660 and 3960, respectively. FFA level was expressed as percent oleic acid, and PV was expressed as millimoles of active oxygen per kilogram of oil. Phenolic compounds were isolated from a solution of oil in hexane by double‐extraction with methanol–water (60:40, v/v). PP content, expressed as tyrosol equivalents (mg kg^−1^ oil), was determined with a UV–visible spectrophotometer (Beckman Coulter, Fullerton, CA, USA) at a wavelength of 735 nm, using Folin–Ciocalteu reagent. Fatty acid composition was determined in the years 2014–2016 by gas chromatograph model 6890 N with flame ionization detection and a DB‐23 capillary column (60 m, 0.25 mm i.d., 0.25 μm film thickness; Agilent Corporation, Santa Clara, CA, USA). Helium, nitrogen and air were employed as carrier gases at a flow rate of 1 mL min^−1^ and the split ratio was 1:50. The injection volume was 1 μL. The initial oven temperature was 165 °C, maintained for 15 min at a heating rate of 5 °C min^−1^ to 200 °C, and then elevated to 230 °C at 25 °C min^−1^ and held at this temperature for 12 min. The temperature of the injector and flame ionization detector was set to 240 °C. The total running time was 35.2 min. The fatty acid peaks were identified using Agilent Technologies software 5988‐5871EN and results were identified by comparing retention times with standard compounds. The relative content of the fatty acid was recorded as peak area percentage. The relative concentrations of eight of the major fatty acids in olive oil were determined: palmitic (C16:0), palmitoleic (C16:1), stearic (C18:0), oleic (C18:1), linoleic (C18:2), linolenic (C18:3), arachidic (C20:0) and eicosenoic (C20:1), which made up over 94% of the total lipids. In addition, the ratios of unsaturated fatty acids (UFA) to saturated fatty acids (SFA) and those of MUFA to PUFA were calculated.

### Leaf and olive paste analysis

Once a year, in July, about 100 diagnostic leaves from non‐bearing shoots of the concurrent year's growth were sampled. The leaves were rinsed once with tap water and twice with deionized water, dried at 70 °C in an oven with forced ventilation, milled to a powder (<0.1 mm), digested with a mix of sulfuric acid and hydrogen peroxide, and the supernatant was analyzed. N and P contents were determined by means of an auto‐analyzer (Lachat Instruments, Milwaukee, WI, USA), and K content was determined with a Perkin‐Elmer AAnalyst 200 atomic absorption spectrophotometer (Milwaukee WI).

A paste sample was taken after crushing the olives and before oil extraction. The sample was dried at 70 °C until its weight stabilized, milled and analyzed for N, P and K contents, using the same procedure described for leaves. Results were presented as percentage of dry matter in both cases.

### Data analysis

Data were analyzed by two‐way analysis of variance (ANOVA) of year and treatment, using JMP software version 14 (SAS Institute, Cary, NC, USA). Significant differences were determined by Tukey–Kramer honest significant difference test at *P* ≤ 0.05.

## RESULTS

**Figure 1:**
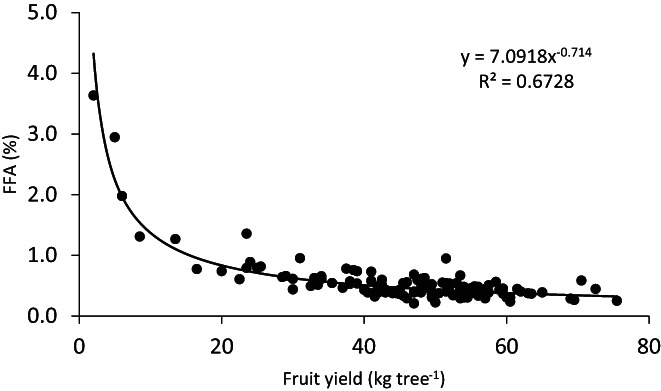
Relationship between FFA content and fruit load. The results are from all fertilization treatments in 2011. Each point represents a single tree.

As found previously,^37^ FFA content of olive oil from cv. Barnea fruit was strongly influenced by fruit load (Fig. 1). When fruit load was lower than 20 kg per tree, FFA increased exponentially, masking the effect of fertilizer level on oil quality. Therefore, in the current study, only data from trees yielding more than 20 kg were considered for the measured quality parameters: FFA and PP contents, PV and fatty acid profile.

### Leaf and olive paste N, P and K concentrations

Table 2 presents leaf and olive paste N, P and K concentrations for 2015. There were significant differences between treatments for all three nutrients, in both leaves and fruit. N and P concentrations were higher in the leaves than in the fruit paste, whereas K concentrations were higher in the fruit paste than in the leaves.

**Table 2:** Leaf and fruit N, P and K concentrations (g kg^−1^ dry matter) in the different treatments (2015 sampling)

Nutrient	N				P		K	
Treatment (kg ha^−1^ y^−1^)	0.45	75	150	300	0	35	0	250
Leaf	12.48c	13.94bc	15.65b	17.93a	0.98A	1.31B	8.53*a*	10.81*b*
Fruit	5.32b	5.58b	6.93ab	7.98a	0.70A	0.87B	11.57*a*	14.47*b*

Numbers followed by different letters differ significantly (Tukey–Kramer, *P* < 0.05).

Some of these results were also presented in Haberman *et al*.^4,5,39^.

### Nitrogen

Two‐way ANOVA revealed no interaction between year and treatment and, therefore, only the treatment effect is presented.

**Figure 2:**
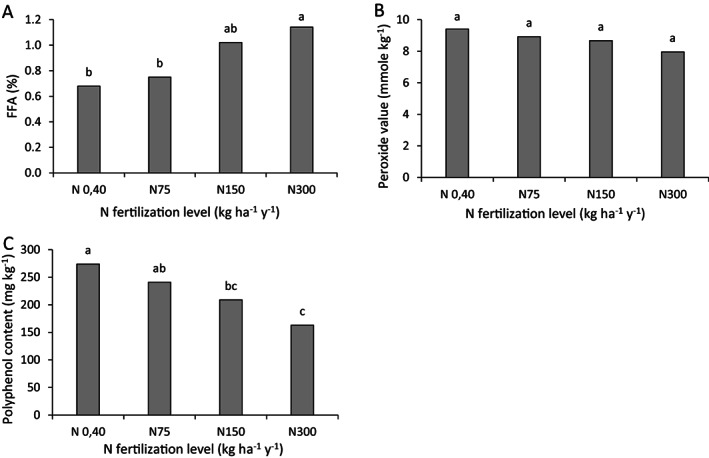
Effect of N fertilization level on (A) FFA content, (B) PV and (C) PP level in olive oil (6‐year average). Columns marked with different letters are significantly different (*P* < 0.05).

The average effect over 6 years of N fertilization level on the olive oil's FFA content, PV and PP content is presented in Fig. 2. FFA level increased significantly with increasing N application (Fig. 2A), from 0.68% (EVOO) at the lowest N application level (N_0,40_) to 1.14% (VOO) with application of 300 kg ha^−1^ y^−1^. PV decreased slightly and consistently with increasing N fertilization (Fig. 2B) but no significant differences were found between treatments. In all cases, PV was within the EVOO range, i.e., below 20 mmol O_2_ kg^−1^.

The highest PP content (Fig. 2C) in the oil was found with the lowest N application level (N_0,40_), 274 mg kg oil^−1^, whereas the lowest PP level, about 163 mg kg oil^−1^, was obtained with the highest N application level.

**Figure 3:**
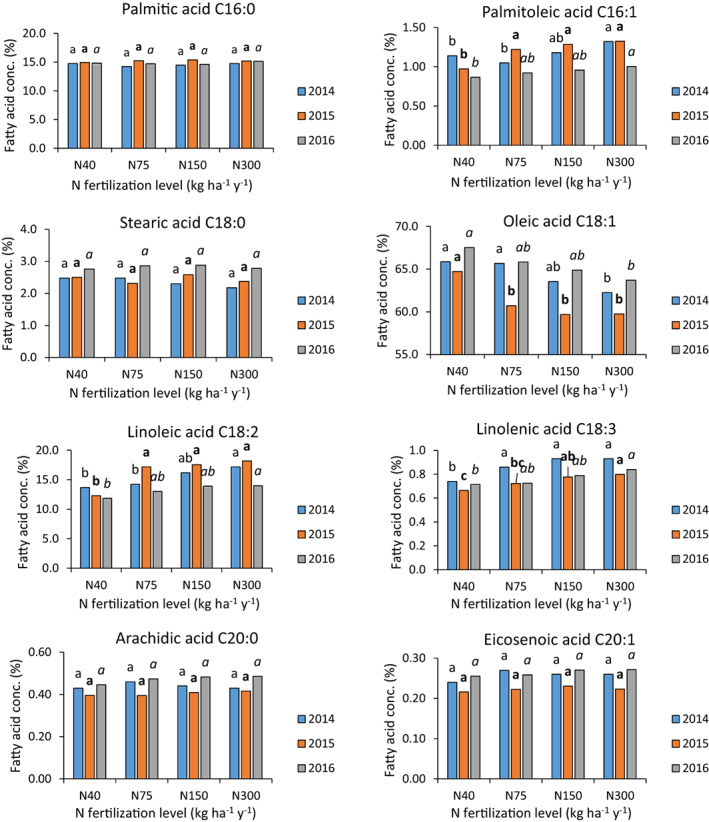
Effect of N fertilization level on fatty acid profile in 2014–2016. Different letters above columns indicate significant differences between treatments in a given year (Tukey–Kramer, *P* < 0.05).

**Figure 4:**
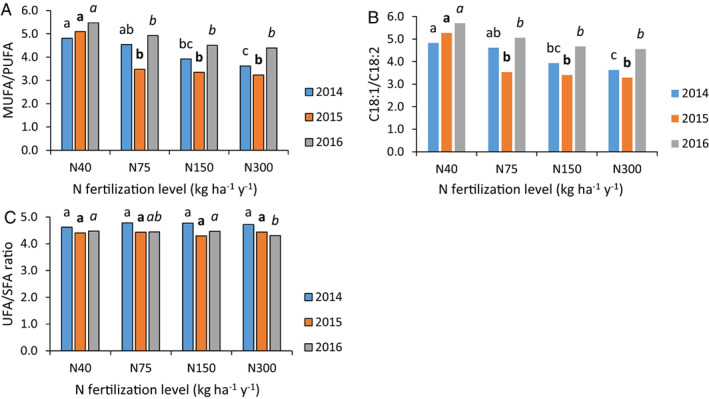
Effect of N fertilization level on MUFA‐to‐PUFA (A), oleic‐to‐linoleic acid (B) and UFA‐to‐SFA (C) ratios. Different letters above columns indicate significant differences between treatments in a given year (Tukey–Kramer, *P* < 0.05).

The relative concentrations of the eight fatty acids measured in this work are presented in Fig. 3. The relative concentrations of palmitic, stearic, arachidic and eicosenoic acids were not significantly affected by N application levels, maintaining the same values throughout the whole range of N levels applied during the years 2014–2016. The concentrations of palmitoleic, linoleic and linolenic acids increased consistently and significantly with increasing levels of applied N. In contrast, the concentration of oleic acid decreased consistently and significantly with increasing N application levels. There were differences in the relative concentrations of the different fatty acids between the years, but the trends remained consistent throughout. MUFA‐to‐PUFA and oleic‐to‐linoleic acid ratios decreased significantly and consistently with increasing N application levels, whereas the UFA‐to‐SFA ratio was not significantly affected (Fig. 4).

### Phosphorus and potassium

FFA content, PV and PP content of the P and K fertilization treatments for the years 2011–2016 are presented in Table 3. In the years 2011–2015, no significant differences were found between treatments for any of the presented quality parameters. However, in 2016, after 5 years of applying the differential treatments, some differences were found for the P treatments: FFA content was significantly higher, and PP content significantly lower, in the P_0_ *versus* P_80_ treatment.

**Table 3:** Oil quality parameters in the P and K treatments applied in the experiment

Quality parameter	Treatment	Year					
		2011	2012	2013	2014	2015	2016
FFA (% oleic acid)	P0	0.385a	1.645a	0.791a	1.177a	1.211a	1.007a
	P80	0.452a	0.999a	1.346a	1.898a	1.465a	0.683b
PV (mmol kg^−1^)	P0	12.3**a**	5.1**a**	6.8**a**	8.4**a**	6.1**a**	6.7**a**
	P80	12.5**a**	5.0**a**	7.9**a**	8.0**a**	5.4**a**	8.2**a**
PP (mg kg^−1^)	P0	301*a*	308*a*	78*a*	105*a*	104*a*	100*a*
	P80	342*a*	252*a*	133*a*	82*a*	129*a*	183*b*
FFA (% oleic acid)	K0	0.480a	0.876a	0.552a	1.930a	1.220a	0.590a
	K300	0.452a	0.999a	1.367a	1.898a	1.465a	0.683a
PV (mmol kg^−1^)	K0	14.3**a**	4.7**a**	8.5**a**	7.9**a**	5.9**a**	8.5**a**
	K300	12.5**a**	5.0**a**	8.2**a**	8.0**a**	5.4**a**	8.2**a**
PP (mg kg^−1^)	K0	249*a*	332*a*	88*a*	80*a*	107*a*	158*a*
	K300	342*a*	252*a*	138*a*	82*a*	129*a*	183*a*

Numbers followed by different letters differ significantly (Tukey–Kramer, *P* < 0.05).

The relative concentrations of the fatty acids measured in this work for the P and K treatments are presented in Table 4. No significant effects of the differential fertilization treatments on oil fatty acid composition, MUFA‐to‐PUFA ratio or UFA‐to‐SFA ratio were found.

**Table 4:** Oil fatty acid profiles, MUFA/PUFA and UFA/SFA relations in P and K fertilization treatments

Treatment	Fatty acid									
	Palmitic acid (%) C16:0	Stearic acid (%) C18:0	Palmitoleic acid (%) C16:1	Oleic acid (%) C18:1	Linoleic acid (%) C18:2	Linolenic acid (%) C18:3	Arachidic acid (%) C20:0	Eicosenoic acid (%) C20:1	MUFA/PUFA	UFA/SFA
P0	14.9	2.2	1.3	63.0	16.5	0.9	0.4	0.3	3.7	4.7
P35/K250	14.5	2.3	1.2	63.5	16.2	0.9	0.4	0.3	3.8	4.8
K0	14.8	2.2	1.3	62.5	17.0	0.9	0.4	0.2	3.6	4.7

## DISCUSSION

As intensive cultivation of oil olives expands, a deeper understanding of the effects of irrigation and fertilization on oil yields and quality is emerging.^26^, ^28^, ^30^, ^31^, ^38^ Macronutrients, especially N, P and K, affect all processes related to olive productivity. When macronutrient levels are increased from deficient to optimal, flowering intensity, fruit set and final fruit yield increase.^4^, ^5^, ^31^, ^39^ The olive tree's response to N, P and K levels is nutrient specific. Over‐fertilization with N reduces both yield and oil quality,^4^, ^29^, ^31^ whereas over‐fertilization with P or K shows no such trend.^5^, ^27^, ^31^, ^39^ Most of the results relating tree nutritional status to oil quality have been obtained from container experiments^28^, ^31^ or those performed under rain‐fed conditions.^29^ Some looked at the combined effects of N, P and K on intensively cultivated olives.^30^ In the current study, the independent effects of N, P and K fertilization on oil quality and composition were studied in a commercial, intensively cultivated olive orchard over six successive years. The effects of the different fertilization treatments on tree vegetative growth, as well as on fruit and oil yields, were described by Haberman *et al*.^4^, ^5^, ^39^

The strong positive relations between N, P and K application levels and their concentrations in leaf and fruit dry matter (Table 2) enabled direct analysis of the relations between oil composition and quality, and N, P and K application rates. In treatments N_0,40_ and N_75_, leaf N concentrations reached deficiency levels after 5 years (12.48 and 13.94 g kg^−1^, respectively, the deficiency threshold value being 14)^29^ and over‐fertilization could be identified in N_300_ with a leaf N concentration of 17.93 g kg^−1^. The P_0_ treatment was on the border of deficiency after 5 years (0.98 g kg^−1^) and the K_0_ treatment did not reach deficiency level. However, P and K leaf concentrations in the P and K fertilized treatments were significantly higher.

### Nitrogen

In Haberman *et al*.,^4^ optimal yields of the olive trees used in the current experiment followed an optimum curve pattern. When N fertilization level was extremely low, low yields were obtained. Increasing N fertilization rates improved yields to an observed maximum, and a further increase in N fertilization above a certain limit (75–150 kg N ha^−1^) was accompanied by yield reduction. Similar results were obtained in a pot experiment carried out using the same olive cultivar.^31^ FFA content, the major olive oil quality indicator, was lowest in the least fertilized treatment and increased with increasing N application levels, in agreement with many other studies.^28^, ^29^, ^40^ FFA content was within the EVOO limits (<0.8%) up to a fertilization level of 75 kg N ha^−1^ y^−1^. Higher N application rates did not increase oil yield^4^ but resulted in higher FFA values (Fig. 2A). Oil PV was not affected by N application rates (Fig. 2B), but its PP content decreased significantly with increasing N application rates (Fig. 2C). PP levels in plant tissues depend on, among other factors, the level of stress exerted on the plant.^41^ In the current study, the lowest N application levels exposed the trees to N stress, leading to higher PP levels. Although olive oil categories do not specify ranges of PP levels, these play an important role as antioxidants, affecting the oil's health properties and stability. These results are in agreement with those found in a container experiment by Dag *et al*.^28^ and Erel *et al*.,^31^ and by Morales‐Sillero *et al*.^40^ in a field trial.

SFA (C16:0, C18:0, C20:0) concentrations were not affected by N application levels. This is in agreement with the results of Dag *et al*.^28^ for irrigated olives grown in containers, but not with Simoes *et al*.,^42^ who found a significant negative correlation between N application level and relative concentrations of SFA in rain‐fed olives. There, leaf N concentrations were always above the threshold value of 1.4% in dry matter. Most UFA concentrations were affected by N application levels. Oleic acid content decreased, while palmitoleic acid, linoleic acid and linolenic acid increased with increasing N levels (Fig. 3). The UFA‐to‐SFA ratio was not affected by N application levels, but MUFA‐to‐PUFA and oleic‐to‐linoleic acid ratios decreased with increasing N. These last two observations are associated with the oxidative stability and health properties of olive oil^43^ and emphasize the importance of adequate and controlled N supply to oil olives. Our results are in agreement with those of Morales‐Sillero *et al*.^30^ and Dag *et al*.^28^ On the other hand, Fernández‐Escobar *et al*.,^29^ examining rain‐fed olives, found no effect of N application level on fatty acid composition. There, the limiting factor was probably water availability and, as presented by others, N concentrations in those orchards were always above 1.4% in leaf dry matter, considered the minimum threshold for adequate N supply. The inconsistent reports regarding the effects of N application level on the fatty acid composition of olive oil^28^, ^29^, ^30^, ^42^ highlight the need for more research to identify additional factors that govern the effect of N, such as cultivar, irrigation, tree nutritional status with other nutrients and environmental conditions.

### Phosphorus

The effect of P application on olive tree performance has been demonstrated only recently.^39^, ^44^ Clear P‐deficiency symptoms in olives are rare^44^, ^45^ and P used to be applied only on the basis of leaf analyses, with 0.1% as a threshold value below which deficiency was considered.^1^, ^34^, ^46^ Furthermore, some studies claimed that olives do not require P fertilization at all, especially if they are grown in soils that are rich in organic matter.^47^ This was based on the presence of large amounts of total P in most soils on the one hand, and the extensive root system of olives coupled with mycorrhizal symbiosis on the other.^48^ Consequently, the number of studies dealing with the effect of P fertilization on olive oil quality and composition is limited. Leaf P concentrations in the plots receiving no P fertilization were below the 0.1% threshold value only in the fifth year of the experiment (Table 2; data from previous years not shown). This is probably due to the large naturally occurring reserves of P in the soil^44^ and the symbiosis between mycorrhizal fungi and olive roots, which improves P uptake.^48^ Apparently, soil P has to be severely depleted before any effect on leaf P concentration can be observed. The effect of P level on FFA and PP contents was found to be statistically significant only in the sixth year of the experiment (Table 3). However, looking at the results of these parameters in the previous years, there are no indications of a developing trend leading to that statistically significant result. Moreover, the response in this work demonstrated a different trend from previous published results from our and others’ works.^28^, ^30^ Dag *et al*.^28^ found a decrease in PP levels and PV with increasing P level, and no response in FFA level. Here, the results of the sixth year indicate a decrease in FFA with higher P, an increase in PP levels with higher P, and no significant response of PV to P application levels. The experiment in Dag *et al*.^28^ was carried out in large containers, with perlite as the substrate. Deficiencies might develop quickly in perlite, which is an inert substrate, whereas in the current experiment the trees were cultivated in the soil, under commercial conditions, and could take up P from natural soil reserves. Moreover, the duration of our experiment was relatively short with regard to the P response. It is suggested that, compared to the relatively rapid response to N levels, P deficiency might appear later in intensively grown olive trees, and only then affect oil quality. Indeed, in the current study, according to the leaf analysis (Table 2), the trees were not exposed to severe P deficiency or over‐fertilization – again, probably due to soil P dynamics and the mycorrhizal symbiosis with olive roots. The latter result, together with the expected effects of P deficiency, calls for continuous monitoring of leaf P levels.

The relative concentrations of fatty acids in the oil in our experiment were not affected by P fertilization levels, in contrast to Dag *et al*.,^28^ who found a decrease in oleic acid and an increase in linoleic and linolenic acid concentrations with increasing P levels. In the same experiment, Haberman *et al*.^39^ found important roles for P in other aspects of olive cultivation, especially those related to productivity. However, we did not find any significant response of oil quality to P fertilization. This indicates that olive productivity is affected by P levels faster than oil quality, and that leaf P concentrations should be far below the 0.1% value in order to affect oil quality. The issue of the effect of available P level on olive oil quality in commercial intensively cultivated olive orchards remains open and additional long‐term experiments are required to obtain a clear answer. Interactions between nutrient availability can have an indirect impact on oil quality. For example, Erel *et al*.^31^ found that high P availability is associated with elevated N uptake.

### Potassium

Of the three macronutrients studied in the current work, K was the only one to show higher concentrations in olive fruit paste compared to leaves (Table 2). The effect of K on tree performance in commercial orchards, both vegetative and reproductive, was reported by Haberman *et al*.^5^ In container experiments, olive trees exposed to a wide range of K levels showed no response in terms of olive oil quality. In the current study, we found similar results: while the effects of K application levels were observed in both leaves and fruit, no effects were found on the assessed oil quality parameters. However, even in the K_0_ treatment, with no supplemental K fertilization for 6 years, leaf K concentrations did not drop below the accepted threshold value for deficiency of 0.8%. This, again, is probably due to the large K reserves in the soil, which become available as K depletion proceeds.^5^ In Dag *et al*.,^28^ no effect of K fertilization on oil quality was found, even with deficient K levels in the trees.

## CONCLUSIONS

Olive oil quality is an important parameter market‐wise as well as health‐wise. We found that in intensive olive orchards N fertilization levels play an extremely important role in the resultant oil quality, whereas P and K are less important. Controlled N fertilization of olive orchards and of many other crops has recently been reviewed, highlighting the vast effects on the environment and food security.^23^ Considering climate change, the impact of decreased water availability and quality and the influence of N fertilization on crop yield and product quality of agronomic crops, an excessive N crop supply might lead to a detrimental environmental impact, reinforcing the need for precise strategies that allow an optimal water and N management adjusted to the specific crop requirements (species, cultivars, and stage of development).

Agricultural soils contain varying amounts of macro elements, and it is therefore difficult to derive general recommendations for fertilization to optimize oil yield and quality. Leaf analysis and, in the future, maybe also fruit analysis should serve as a useful diagnostic tool for tree nutritional status and the required corrective fertilization steps, especially as olive cultivation expands to new agricultural regions and soils.

## CREDIT AUTHORSHIP CONTRIBUTION STATEMENT

**IZ:** conceptualization, methodology, formal analysis, investigation, writing. **UY:** conceptualization, methodology, investigation, funding acquisition, writing – review and editing. **AD:** conceptualization, investigation, writing – review and editing. **RE:** conceptualization, investigation, writing – review and editing. **ABG:** conceptualization, investigation writing – review and editing. **LQ:** investigation, chemical analysis, data analysis, writing. **ZK:** conceptualization, investigation, writing – review and editing.

## CONFLICT OF INTEREST

The authors declare no conflicts of interest.
